# Comparison of the urinary microbiome in men who have sex with men with and without *Chlamydia trachomatis* infection

**DOI:** 10.1007/s10096-024-04930-8

**Published:** 2024-09-11

**Authors:** Kehinde C. Mofolorunsho, Nonkululeko G. Mabaso, Nikita Nundlall, Abidemi O. Ojo, Errol D. Cason, Nathlee S. Abbai

**Affiliations:** 1 School of Clinical Medicine Laboratory, College of Health Sciences, University of KwaZulu- Natal, Durban, South Africa; 2 Centre for Applied Food Sustainability and Biotechnology (CAFSaB), Central University of Technology, Bloemfontein, South Africa; 3 Department of Animal Science, University of the Free State, Bloemfontein, South Africa

**Keywords:** MSM, Sexually transmitted infections, Urinary microbiota, *Prevotella*, South Africa

## Abstract

**Purpose:**

The urinary tract is colonized by microbial communities that impact urinary health. Previous studies have suggested that the bacterial composition of the male urinary microbiota is related to STIs. This study assessed the bacterial composition of the urinary microbiome in South African MSM with and without *C. trachomatis*.

**Methods:**

This study used urine samples from MSM attending care at the King Edward VIII hospital and the Aurum Institute in Durban, South Africa. A total of 200 samples were tested for *C. trachomatis* infection using the Applied Biosystems™ TaqMan^®^ Assays. Urinary microbiomes of 23 samples were characterized using 16 S rRNA (V3 and V4) gene sequencing on the Illumina MiSeq platform.

**Results:**

Bacterial taxonomic analysis showed a high abundance of *Streptococcus*, *Corynebacterium*, and *Staphylococcus* in all the sequenced samples. Moreover, *Prevotella* and *Lactobacillus* were detected in urine samples of MSM. Alpha diversity metrics showed a slight increase in microbial diversity in *C. trachomatis* positive samples; however, this was not significant (ANOVA, *P* > 0.05). Principal coordinates analysis (PCoA) showed that the microbiome of *C. trachomatis* infected MSM was not clearly different from those uninfected. Distinct bacterial communities were not detected between positive and negative samples (PERMANOVA F_1,22_= 1.0284, R^2^ = 0.047%, *P* = 0.385).

**Conclusion:**

Most microbiome studies on MSM to date have focused on the gut microenvironment. Few studies, however, have provided data regarding the normal composition of the male urethral microbiomes or if these microbiomes are associated with male STIs. This study adds to the growing body of knowledge highlighting the urinary microbiome in MSM.

**Supplementary Information:**

The online version contains supplementary material available at 10.1007/s10096-024-04930-8.

## Introduction

The human microbiome consists of approximately 38 trillion commensal and pathogenic microorganisms existing in the human body [1, 2]. The human body harbours complex microbial communities ranging from bacteria and eukaryotic viruses, to protozoa and fungi. These microorganisms are known to coexist with human organisms and are found in several body sites such as the skin, nasal tract, respiratory tract, gastrointestinal tract and urogenital tract [1, 3–6].

The human microbiome has been shown to contribute to the human condition [1], ensuring vital functions for the host and may affect health and disease susceptibility by regulating physiological homeostasis, energy metabolism, and immune-related bioprocesses [4, 6]. This community of microorganisms plays an important role in maintaining health [7]. However, changes in the microbiome or dysbiosis can result in the development of several pathological conditions such as type II diabetes mellitus [8], Crohn’s disease [9], non-alcoholic fatty liver disease [10], allergies [11], inflammatory bowel disease [12] and bacterial vaginosis [13].

Although comprehensive studies investigating the human microbiome have generated an unprecedented understanding of the microorganisms that colonize the human body [14], not all body sites were considered suitable for initial examination [15]. The urinary tract was initially thought to be sterile [16–19] and was not included in early microbiome research [17]. However, the existence of a urinary microbiome has been acknowledged over the last decade [19–21]. With the advent of evolving sensitive tools such as next-generation sequencing [16], the characterization of urine samples from human bladders discovered unique bacterial communities that make up the urinary microbiome [18, 19, 22].

Rich and complex microbial communities are present in the human urinary tract, and alterations in its composition (dysbiosis) are thought to be implicated in varying diverse urinary tract symptoms and disorders [23, 24]. Research has demonstrated that the urinary microbiome influences the risk for the development of urinary tract infections (UTIs), urinary incontinence, and persistent lower urinary tract symptoms in women [22, 25, 26]. Nevertheless, little is known about the urinary microbiome of males, especially those of men who have sex with men (MSM) [27]. The microbiome of the male urogenital tract is poorly described. On the other hand, it has been proposed that bacterial colonization of the male urethra may influence the risk of sexually transmitted infections (STIs) [7] including *Chlamydia trachomatis* (*C. trachomatis*).

*C. trachomatis* is an obligate intracellular bacterium that causes the disease chlamydia [28, 29]. Following infection with *C. trachomatis*, the pathogen migrates into the female upper genital tract [28–30], where it infects the cervix and urethral columnar epithelial cells, resulting in uncomplicated cervicitis and urethritis [31]. In men, this pathogen infects susceptible transitional epithelial cells which leads to urethritis [28]. Several studies have found that the genital microbiome plays a role in the host’s susceptibility to chlamydial infection [7, 32–35].

Microbiome variation has been found to be significantly influenced by sexual practice. MSM exhibit a distinct microbiome signature characterized by *Prevotella* enrichment and increased alpha diversity, which is linked with receptive anal intercourse in both males and females [36]. It is assumed that certain genital microbiomes may cooperate with *C. trachomatis* to absorb indole from *Prevotella*, allowing the bacteria to evade the inhibition of IFN-γ [35, 37]. MSM are a vastly understudied population at high risk for genitourinary diseases such as chlamydia and other STIs [27, 38, 39]. The urethra, rectum and pharynx are the common sites of STIs among this key population due to unprotected oral/anal sexual behaviors [38]. Exposure to the gut and oral microbiome through these routes may lead to shifts in the urinary microbiome of MSM that increase predilection for genitourinary diseases [27]. The majority of microbiome studies on MSM to date have focused on the gut microenvironment [40–42]. Few studies, however, have provided data regarding the normal composition of the male urethral microbiomes or if these microbiomes are associated with male STIs [7, 27, 43]. Hence, our study aimed to assess the bacterial composition of the urethral microbiome in a cohort of MSM with and without *C. trachomatis* infection.

## Materials and methods

### Ethics statement

Ethical approval for this study was obtained from the Biomedical Research Ethics Committee (BREC) of the University of KwaZulu-Natal (BREC/00002798/2021).

### Study population

This cross-sectional study used urine samples from sexually active MSM, 18 years and older, and attending care at the King Edward VIII Hospital and the Aurum Institute, both located in Durban, South Africa. All laboratory assays were conducted at the School of Clinical Medicine Research Laboratory of the Nelson R. Mandela School of Medicine, University of KwaZulu-Natal.

### Sample processing and DNA isolation

There were 200 available urine samples from study participants enrolled between October 2021 and July 2022. These samples were refrigerated between 2 and 8 °C until processed. A total of 10 ml of each urine was centrifuged at 14,000 rpm for 45 min, and the supernatant discarded. The recovered pellets were then subjected to further molecular analyses. Deoxyribonucleic acid (DNA) was extracted from the sample pellets using the PureLink Microbiome Kit (ThermoFisher Scientific, United States), following the manufacturer’s protocol, and quantified using the Nanodrop Spectrophotometer (ThermoFisher Scientific, United States). The extracted DNA was stored at − 20 °C until subsequent analysis.

### Detection of *C. trachomatis*

*C. trachomatis* was detected using the Applied Biosystems^™^ TaqMan^®^ Assays. Commercial primers and probes (Ba04646249_S1) which targets the translocated actin-recruiting phosphoprotein of *C. trachomatis*, were used. Amplification was performed on the Quant Studio 5 Real-time polymerase chain reaction (PCR) detection system (Thermo-Fisher Scientific, United States). Each reaction was performed in a final volume of 10 µl and included: 1 µl FAM-labelled probe/primer mix for individual targets, 5 µl Fast Start 4x probe master mix (Thermo-Fisher, Ba04646249_S1), 1.5 µl template DNA and 2.5 µl nuclease-free water. The runs included non-template control reactions. Amplification was performed under the following conditions: 1 cycle at 95 °C for 30 s followed by 45 cycles of denaturation at 95 °C for 30 s and annealing at 60 °C for 30 s. At the end of the annealing period, samples with fluorescence were deemed positive for chlamydia.

### Illumina 16 S rRNA gene sequencing

The V3 – V4 hypervariable regions of the 16S ribosomal ribonucleic acid (rRNA) gene from urine genomic DNA (gDNA) were amplified using forward primer 5’-TCG TCG GCA GCG TCA GAT GTG TAT AAG AGA CAG CCT ACG GGN GGC WGC AG- 3’ and reverse primer 5’-GTC TCG TGG GCT CGG AGA TGT GTA TAA GAG ACA GGA CTA CHV GGG TAT CTA ATC C-3’ [44, 45]. Reactions were performed in a total volume of 25 µL, consisting of 25 ng gDNA template, 1.5 µL (0.3 µM) of each primer, 12.5 µL KAPA HiFi HotStart Ready-mix (1X) (ROCHE, Randburg, South Africa) and 7 µL nuclease-free water. The DNA library was prepared using the PCR products, following the Illumina TruSeq^®^ Nano DNA Sample Preparation protocol. Sequencing was performed on an Illumina MiSeq, using a 2 × 301 cycle paired-end approach. The sequencing data have been deposited in the National Centre for Biotechnology Information (NCBI) BioProject PRJNA1089515 with the Biosample accessions SAMN40541621- SAMN40541643.

### Data processing and statistical analysis

The 16 S rRNA gene sequences obtained from Illumina sequencing were analyzed using the QIIME 2 pipeline [46]. Sequencing remnants and adapter sequences were removed using Cutadapt [47]. The paired-end sequences were imported into QIIME 2, quality-controlled and combined using DADA2. DADA2 first truncated both the forward and reverse sequencing reads at positions in the reads supplied. These positions were determined based on a visual inspection of the quality distribution of the reads. Sequences were grouped into amplicon sequence variants (ASVs) based on 99% sequence similarity. The ASV table was built, chimeras were removed, and the taxonomy for the 16 S rRNA reads was assigned using the SILVA 132 [48] database. For taxonomic classification, the q2-feature-classifier plugin was used to train naïve Bayes classifiers on reference data sets using Scikit-learn. Alpha rarefaction curve was plotted with 1000 sampling depths.

The processed gene sequence data was further analyzed using RStudio, an integrated development environment (IDE) for the programming language R [49]. The following R packages were used during the processing of the taxonomic data: Phyloseq [50], Tidyverse [51], Vegan [52], and Biomformat [53].

For beta diversity analysis, the taxonomic structures of the microbial communities were visualized using principal coordinate analysis (PCoA). A permutational analysis of variance (PERMANOVA) was used to assess beta diversity differences between different sampled groups. These analyses were performed with the “adonis” functions in vegan for R.

## Results

In the current study, a total of 200 MSM were tested for *C. trachomatis* infection. Of the 200 men, 12 (6.0%) tested positive for *C. trachomatis* (Table 1). Therefore, to have a balanced number of samples for analysis, the same amount of samples which tested negative for *C. trachomatis* infection were randomly selected. Thus a total of 24 samples, 12 positive and 12 negative were selected for sequencing. However, one of the 12 positive samples was excluded from microbiome analysis, as the sample had insufficient DNA. Therefore, a total of 23 samples were sequenced.

**Table 1:** Characteristics of the study population according to *C. Trachomatis* infection status

	C. trachomatis Negative	C. trachomatis Positive	Total	*p*-value
	188 (94.0%)	12 (6.0%)	200	
**Age group (years old)**				0.037
18–24	21 (11.1%)	3 (25.0%)	24 (12.0%)	
25–30	75 (39.9%)	8 (66.7%)	83 (41.5%)	
31–44	78 (41.5%)	1 (8.3%)	79 (39.5%)	
45+	14 (7.5%)	0 (0.0%)	14 (7.0%)	
**Has symptoms of STIs**				0.738
No	92 (48.9%)	5 (41.7%)	97 (48.5%)	
Yes	88 (46.8%)	7 (58.3%)	95 (47.5%)	
Refused to answer	8 (4.3%)	0 (0.0%)	8 (4.0%)	
**HIV status**				0.969
Negative	121 (64.4%)	8 (66.7%)	129 (64.5%)	
Positive	53 (28.1%)	3 (25.0%)	56 (28.0%)	
Did not know status	14 (7.5%)	1 (8.3%)	15 (7.5%)	

High-throughput sequencing yielded between 291 and 249,759 reads for the twenty-three samples. After quality filtering, denoising, merging and chimera filtering between 5 and 86,943 sequences remained (Table S1). Rarefaction curves suggested that the sequencing depth was adequate to capture most of the prokaryotic diversity in most of the samples (Figure S1).

**Fig. 1:**
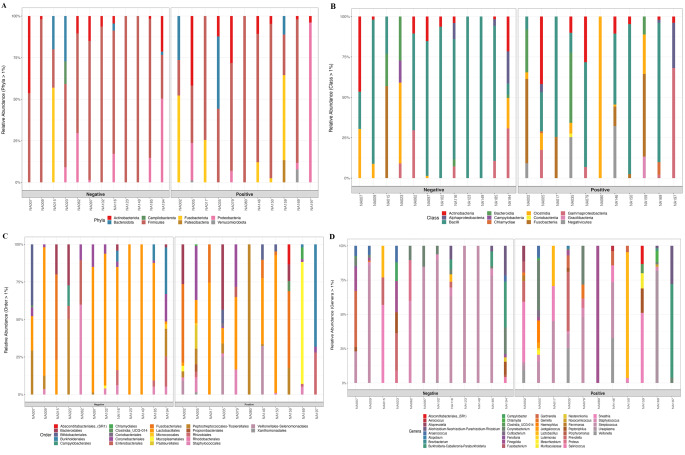
Taxonomic information based on 16 S rRNA gene sequences. Stacked bar charts of taxonomy relative abundances at phylum (**A**), class (**B**), order (**C**) and genus (**D**) level for the *C. trachomatis* negative and *C. trachomatis* positive group

Based on phylogenetic classification using the SILVA 16 S rRNA gene database, sample diversity at the phylum level classified most reads as *Firmicutes*, *Actinobacteriota*, *Proteobacteria*, *Bacteroidota* and *Fusobacteriota* (Fig. 1A; Table S2). Sample diversity at the genus level identified a diverse consortium in all samples (Fig. 1D; Table S2). Some of the more dominant genera in the samples included *Streptococcus*,* Corynebacterium*,* Staphylococcus*, *Prevotella*, *Gardnerella*, and *Lactobacillus*. In contrast, sequences corresponding to *Chlamydia*, *Alloprevotella*, and *Parvimonas* were present in lower abundances.

**Fig. 2:**
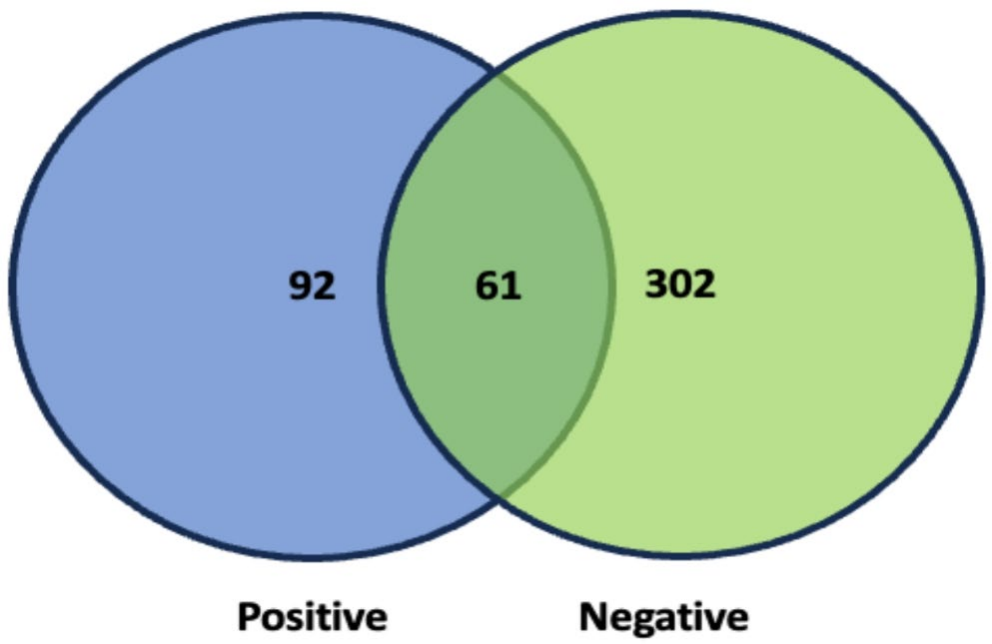
Venn diagram showing the number of unique and shared ASVs between samples from *C. trachomatis* infected (blue) and uninfected (green) groups

A total of 455 amplicon sequence variants (ASV) were identified across all samples. Of the total number of ASVs, 92 (representing 5.92% of the total number of sequences) were unique to the positive samples, 302 (16%) were unique to the negative samples and 61 (78.08%) were shared between both (Fig. 2).

**Fig. 3:**
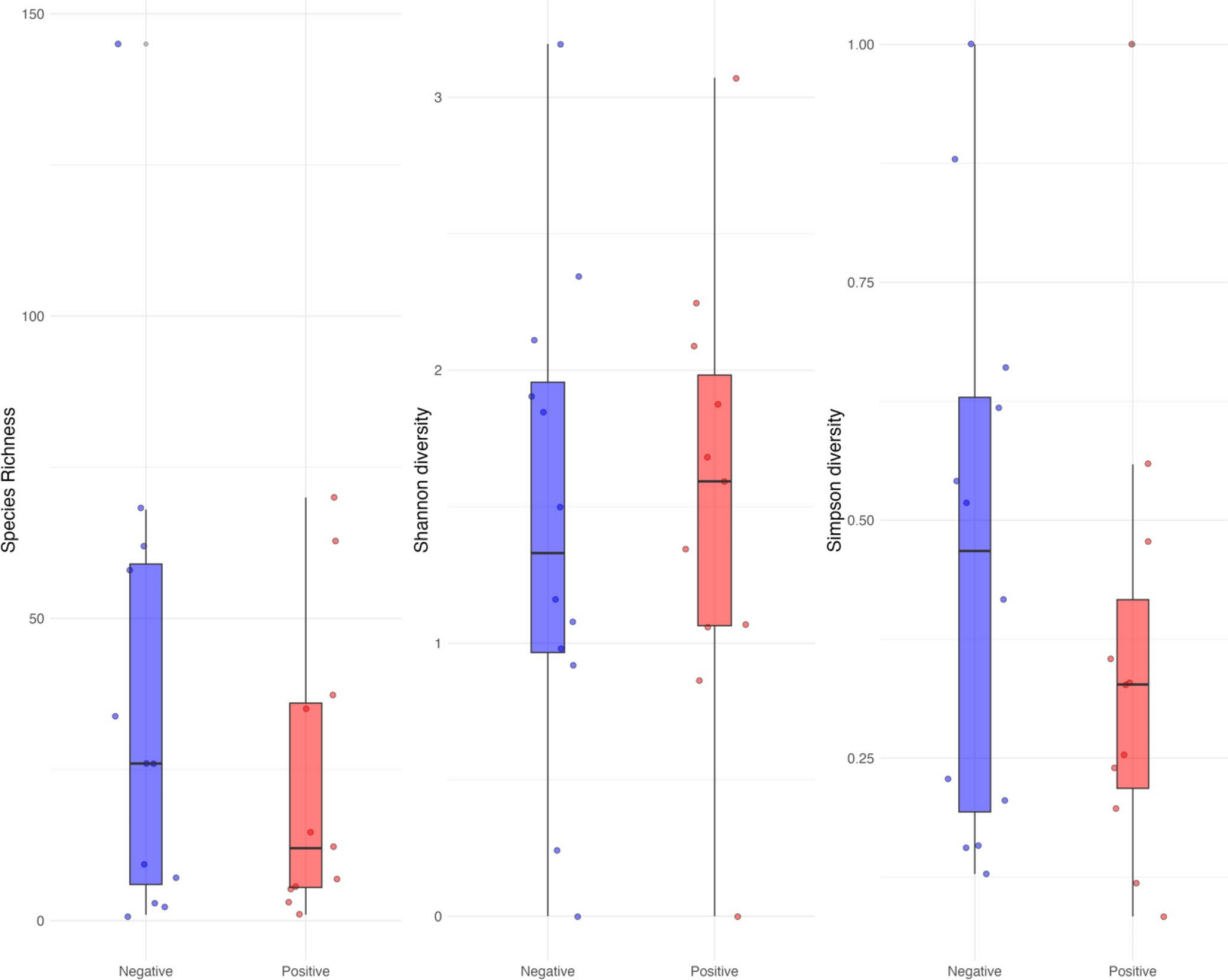
Alpha diversity boxplots of Richness, Shannon and Simpson indexes. Diversity measures of microbial species recovered from urine samples were not different between *C. trachomatis* positive and *C. trachomatis* negative groups

Alpha diversity metrics (Richness, Shannon and Simpson) showed a slight increase in microbial diversity in positive samples; however, this was not significant (ANOVA, *P* > 0.05) (Fig. 3).

**Fig. 4:**
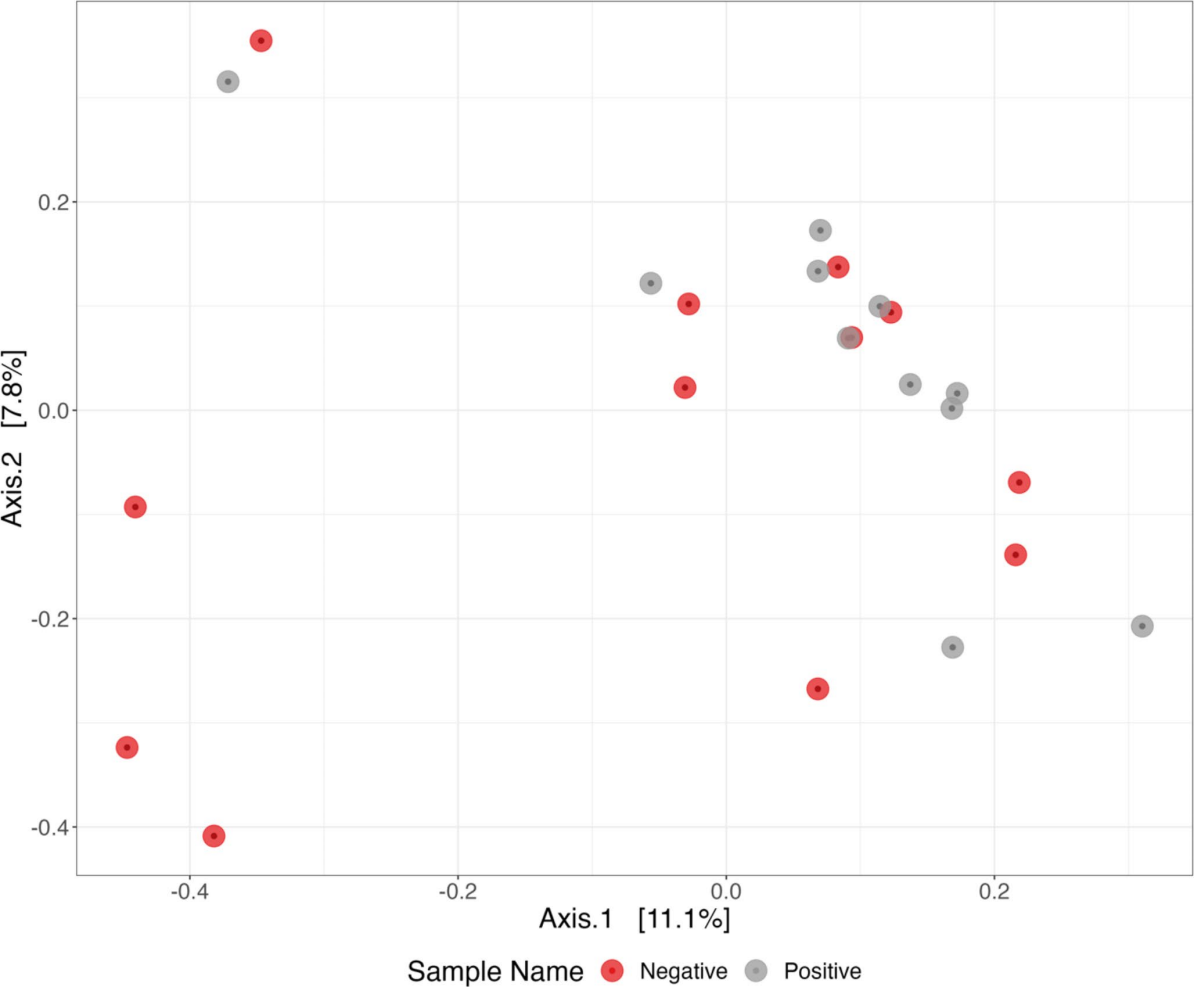
Principal Coordinates Analysis (PCoA) plot showing structural differences between bacterial communities based on unweighted Unifrac dissimilarity matrix. The grey dots indicate urine samples from MSM positive for *C. trachomatis* and the red dots correspond to urine samples from MSM negative for *C. trachomatis*. The percent variations explained by each principal coordinate are shown in parentheses

Principal coordinates analysis (PCoA) showed that the microbiome of *C. trachomatis* infected MSM was not clearly different from those uninfected. Overall, whether samples tested positive or negative was not important in explaining structural differences among bacterial communities (Fig. 4). Distinct bacterial communities (Operational Taxonomic Unit level) were not detected between positive and negative samples (PERMANOVA F_1,22_= 1.0284, R^2^ = 0.047%, *P* = 0.385) using normalized unweighted UniFrac dissimilarities.

**Fig. 5:**
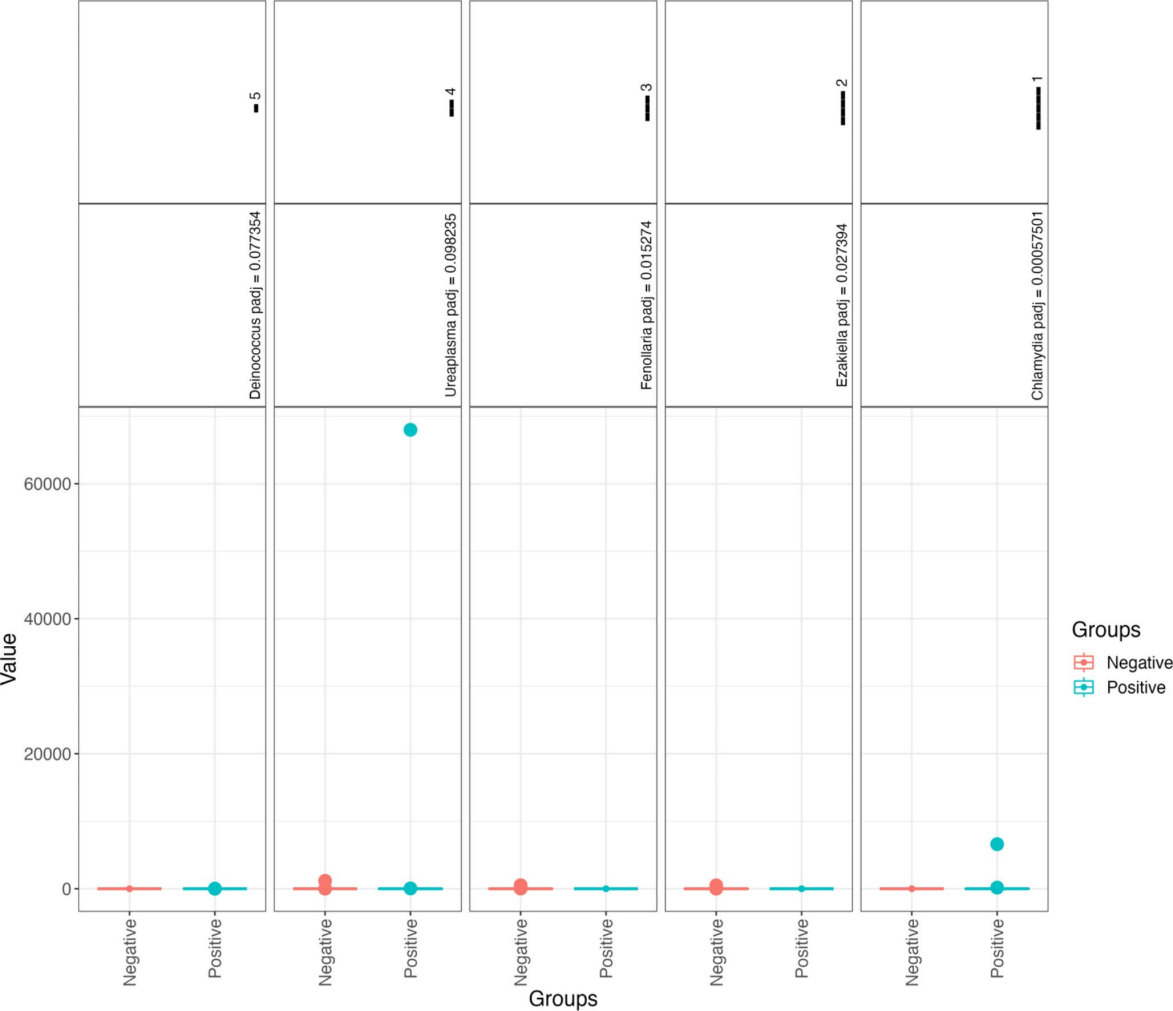
Presence-absence analyses identified only 1 genus with a significantly different probability of presence between MSM with and without *C. trachomatis*

Presence-absence results differed between MSM with and without *C. trachomatis* in 1 of the 5 groups analysed. The genera *Chlamydia* (padj = 0.001) had higher probabilities of presence in *C. trachomatis* positive MSM (Fig. 5).

**Fig. 6:**
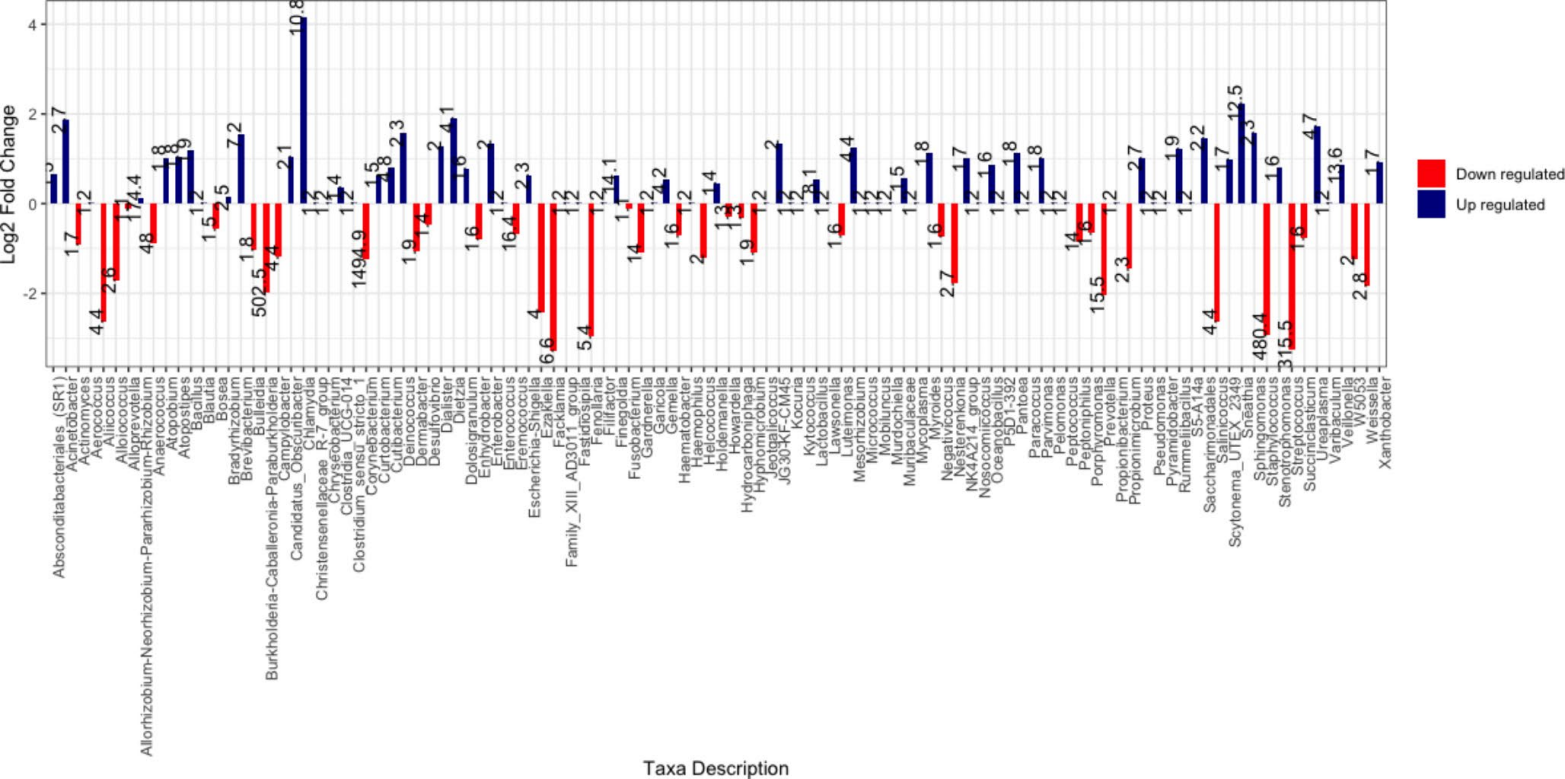
Log2 fold change in abundance in positive samples compared to negative samples of other bacterial taxa identified in urine samples

Figure 6 shows the log2 fold change (log2FC) of other identified bacterial taxa constituting the urine microbiome of MSM. A positive log2FC means a higher abundance for that taxa in the positive samples, while a negative log2FC means a lower abundance for that taxa. Of the 108 abundant taxa, 37 (red) were less abundant, and 71 (blue) were more abundant in the positive samples.

## Discussion

*Chlamydia trachomatis*, a common sexually transmitted infection (STI), is the most reported bacterial STI in the world. It is considered a public health problem due to the high occurrence of active infection in women and men, including MSM [54–56]. Currently, around 131 million people worldwide are infected by *C. trachomatis* each year [57]. To the best of our knowledge, there is a lack of information in South Africa on the bacterial microbiota in urine from MSM with and without *C. trachomatis*. In this study, the urinary microbiome in the MSM with and without *C. trachomatis* was investigated.

The male genital microbiota has not been as thoroughly researched as the cervicovaginal microbiota. A few of these studies have used urine samples, which may be reflective of the urethral microbiota [58]. Some studies have also characterized using next-generation sequencing (NGS), the urine and urethral microbiota in asymptomatic men [59–61] and reported a diverse bacterial microbiota which were highly variable from person to person. Similarly, our study revealed a high diversity, as study participants harboured diverse taxons. This is in consonance with findings from a previous study, which reported that a large diversity of microbial species were present in the urinary microbiota of MSM [27]. Another study found that the male urethral microbiota contains a very diverse composition of bacteria, both in patients and in healthy controls [62]. In contrast, a study by Dong et al. [61] found fewer genera. Dong et al. found 88 genera in samples from 10 men positive for *C*. *trachomatis*, *N*. *gonorrhoeae* or *T*. *vaginalis*, and 131 genera in the 22 STI-negative men [61]. Our finding also supports prior studies indicating that the male urinary tract harbours a complex bacterial community that is distinct from other body microenvironments [63–65].

In this study, bacterial taxa were identified and classified to the genus level. The analysis of taxonomic classification at the phylum level showed that most samples were typically composed of five major phyla. Sequences corresponding to five bacterial phyla including *Firmicutes*,* Actinobacteriota*,* Proteobacteria*,* Bacteroidota* and *Fusobacteriota* were frequently detected, whereas sequences corresponding to *Verrucomicrobiota* were less abundant. Differences in the distribution of these phyla among samples were also observed. Firmicutes were present in 22/23 of the urine samples, while *Actinobacteriota* and *Proteobacteria* were identified in 15/23 and 11/23 urine samples, respectively. *Bacteroidota* and *Fusobacteriota* were present in 7/23 and 6/23 urine samples. However, *Verrucomicrobiota* were detected in only 2/23 urine samples. A study by Nelson and colleagues on urine microbiomes and asymptomatic STIs in men identified the following bacterial phyla: *Firmicutes* (52.6%), *Actinobacteria* (18.7%), *Fusobacteria* (10.0%), *Proteobacteria* (9.4%) and *Bacteroidetes* (7.4%). The authors of this study also reported that urine from sexually active men often contains complex microbial communities and that the composition of these urine communities is relevant to STIs [7]. In another published study that characterized the urinary microbiome of 10 healthy men using the 16 S rRNA sequencing-based methods, bacterial DNA sequences taken from the urine of study participants were predominantly *Firmicutes* (65%). Other predominant phyla included *Actinobacteria* (15%), *Bacteroidetes* (10%) and *Proteobacteria* (8%) [66]. Another study found that *Bacteroidetes*, *Firmicutes*, and *Proteobacteria* were the most abundant phyla in three types of urine samples from a population of men [67]. The observed differences in phyla distribution from this current study support the results from a previous study, which indicated that there is substantial intra-individual variation in urine microbiomes at the phylum level [7].

The analysis of the urinary microbiota composition at the genus level showed high abundance of *Streptococcus*,* Corynebacterium*, and *Staphylococcus* in both the *C. trachomatis* infected and uninfected groups. In addition, *Prevotella*, *Gardnerella*, and *Lactobacillus* were detected in the urethral microbiota of MSM in this study. Other identified genera included *Sneathia* and *Veillonella*, which were less abundant. Consistent with our findings, Sawhney et al. also recovered similar organisms in a cohort of asymptomatic MSM: *Gardnerella*,* Streptococcus*,* Staphylococcus*,* and Corynebacterium* [27]. In a prior study involving males visiting a STI clinic, *Lactobacillus*, *Corynebacteria*, and *Streptococcus* were among the most frequently detected genera. That same study also showed that STIs were associated with organisms not usually related to the male urinary tract, such as *Sneathia* and *Prevotella* [7]. Other studies have also detected similar genera in urine samples from their population of men, including *Prevotella*, *Veillonella* [61, 67], *Lactobacillus*, and *Sneathia* [61]. Findings from our study support that commonly recovered genera in the female urinary microbiota are similar to those detected in MSM [27] and that the urogenital microbiota overlap to a larger extent with vaginal and other mucosal-associated organisms as well as skin-associated microorganisms [35, 68].

Urotypes such as those dominated by *Prevotella* [70], and *Lactobacillus* [43, 63, 65, 66] in the female urinary microbiota have been described as members of the male urinary microbiota [43, 63, 65, 69, 70]. Previous studies have consistently reported that the proportion of *Lactobacillus* in the male urinary microbiota is lower when compared to females [43, 63, 66]. Our results support this observation as only 5 of the 23 urine samples in our investigation had *Lactobacillus* detected. Similarly, a study that assessed the composition of the urinary microbiota of MSM reported that *Lactobacillus* was recovered in only two of 129 urine samples [27].

Changes in the urinary microbiota of MSM may result from exposure to unique microenvironments, such as the gut and oral microbiota, through anal and oral sex [27]. Similarly, it has been demonstrated that the rectal microbiota of MSM who engage in condomless receptive anal intercourse, is enriched for the family *Prevotellaceae* [41]. The gut microenvironment has been the subject of the majority of MSM microbiome research to date. These studies have shown that the gut microbiome of MSM is enriched in *Prevotella* when compared to non-MSM populations [27, 40–42]. In the present study, we detected *Prevotella* from urine samples in both the MSM with and without *C. trachomatis* groups. This finding is in agreement with that of Sawhney et al. who also found that *Prevotella* was one of the most frequently recovered genera from urine samples in MSM. Their finding also suggested a potential gut reservoir as the source of *Prevotella* in the urinary microbiota, with the recovery of *Prevotella* in 59% of MSM who reported recent insertive anal sex [27]. Nelson et al. [7] have demonstrated that the presence of *Prevotella* in urine samples positively correlates with STIs in men and that men with asymptomatic STIs such as *C. trachomatis* were more likely to have urine microbiota dominated by fastidious, anaerobic, and uncultivated bacteria than those without STIs [7].

Our study also identified other genera commonly found in healthy men (*Streptococcus*, *Staphylococcus*, *Gardnerella*, and *Corynebacterium*), including those (*Sneathia* and *Veillonella*) observed in the vaginal microbial communities of women with bacterial vaginosis (BV) [71, 72]. Similar to our findings, these genera including *Veillonella*, were among the microorganisms recovered in a study involving a cohort of healthy MSM [27]. Manhart et al. in their study, found *Sneathia* spp., which is commonly present in BV to be associated with male idiopathic urethritis [73]. Another study, which examined urine in males visiting a STI clinic without symptoms of urethritis, found that *Sneathia spp*. were the dominant components of the urine microbiome [7]. Several studies have also shown that the male urinary microbiota is dominated by bacteria such as *Corynebacterium* [74], *Gardnerella* [27, 63], *Staphylococcus* [75, 76], and *Streptococcus* [27, 63, 66].

*Corynebacterium* was one of the commonly detected bacteria in MSM with and without *C. trachomatis* in our study. *Corynebacterium* is frequently recovered from male urine as well as urethral samples [43 60, 62, 77]. *Corynebacterium* is generally considered a commensal of the male genital microbiota [78] and has been associated with positive health outcomes [79]. However, since *Corynebacterium* is a diverse genus, it is possible that specific *Corynebacterium spp*. account for a small number of urethritis cases [79]. A couple of case reports have associated specific species of *Corynebacterium* with urethritis [80–82]. Therefore, further studies on the role of *Corynebacterium spp*. in infection may be relevant to understanding a variety of urogenital disorders in male populations and MSM as well.

*Gardnerella* has been shown to be present in the vaginal microbiome, with certain species thought to play a key role in BV [83]. However, this genus has been associated with urethritis in men [84, 85]. *Gardnerella* was recovered in this current study. A study conducted among men with and without idiopathic urethritis showed that 28% of MSM who reported not having a female sexual partner three months prior to enrolment had *Gardnerella* present in their urethral microbiota [79]. Other studies have reported that BV-associated bacteria were detected in the genital microbiome of male partners of women without BV [86, 87]. Hence, it is plausible that BV-associated bacteria including *Gardnerella*, are not entirely acquired from the vagina but may form part of the autochthonous male urethral microbiota or may be present in the rectum or mouth [79].

A limitation of this study is that DNA libraries generated for the target metagenomics for this study did not generate adequate sequencing data due to the low number of sequences. Also, the sample size used in the study was small. Future studies with a larger sample size will provide more clarity on the role of urinary microbiomes in MSM sexual health and non-gonococcal urethritis (NGU).

In conclusion, this study adds to the growing literature highlighting the urinary microbiome in MSM. Urine samples from MSM with and without *C. trachomatis* infection was shown to contain a diverse microbiota. We identified several specific bacterial taxa associated with this key population and also showed that BV-associated genera, such as *Gardnerella* and *Sneathia*, were components of the urethral microbiota of MSM. It is clear from our study that unique microbial communities, especially those associated with the female urinary tract, are present in MSM. Therefore, continued research into the role of the urinary microbiota in urinary health of this understudied population is required. This will provide clarity as to whether these microorganisms impact risk for STIs.

## Electronic supplementary material

Below is the link to the electronic supplementary material.

**Supplementary Material 1**: **Figure S1**. Rarefaction curve showing species richness in association with sample sequences. Supplementary Table S1. Denoising statistics. Supplementary Table S2. Relative abundance at the genus level. **Table S1.** Denoising statistics. **Table S2.** Relative abundance at genus level.

## Data Availability

Authors confirm that sequence data supporting the findings of this study have been deposited in the National Centre for Biotechnology Information (NCBI) BioProject PRJNA1089515 with the Biosample accessions SAMN40541621- SAMN40541643. Other relevant data that support the findings of this study are included in the manuscript or uploaded as supplementary information.
