# Supplementary material for: Comparison of the urinary microbiome in men who have sex with men with and without Chlamydia trachomatis infection
Source: Eur J Clin Microbiol Infect Dis. 2024 Sep 11;43(11):2159–70. doi: 10.1007/s10096-024-04930-8 (PMC11534976; doi:10.1007/s10096-024-04930-8)
Supplement: Supplementary file 1 — Supplementary Material 1: Figure S1. Rarefaction curve showing species richness in association with sample sequences. Supplementary Table S1. Denoising statistics. Supplementary Table S2. Relative abundance at the genus level. Table S1. Denoising statistics. Table S2. Relative abundance at genus level. [file 10096_2024_4930_MOESM1_ESM.docx]

**Supplementary Material
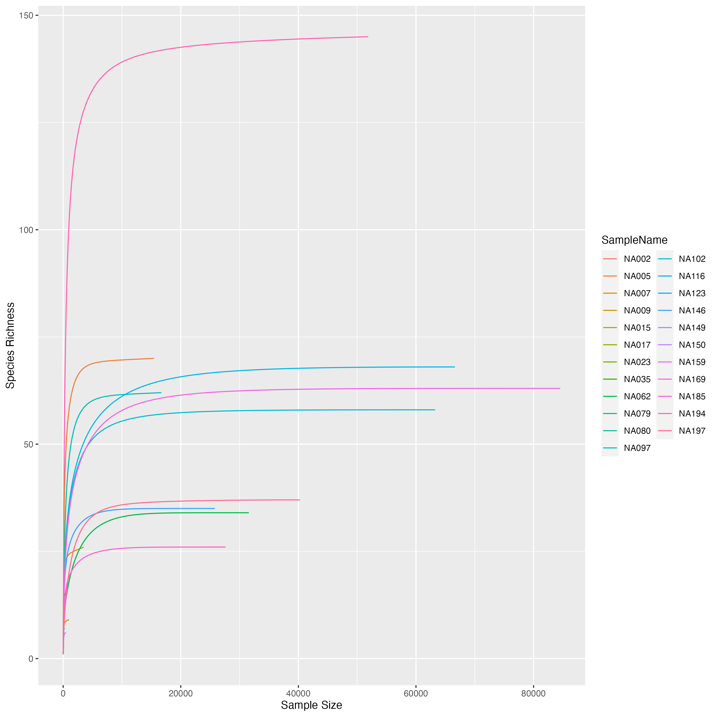
**

**Figure S1.** Rarefaction curve showing species richness in association with samples sequences

**Table S2.** Relative abundance at genus level
